# Introduction to the CLEM technique developed in the field of neuroanatomy

**DOI:** 10.1007/s12565-025-00875-w

**Published:** 2025-07-23

**Authors:** Takaichi Fukuda

**Affiliations:** 1 Department of Anatomy and Neurobiology, Graduate School of Medical Sciences, Kumamoto University, Kumamoto, 860-8556 Japan; 2 Center for Metabolic Regulation of Healthy Aging, Faculty of Life Sciences, Kumamoto University, Kumamoto, 860-8556 Japan; 3 Center for Clinical Laboratory, Kumamoto Rosai Hospital, 1670 Takehara-Machi, Yatsushiro, Kumamoto, 866-8533 Japan

**Keywords:** Immunoelectron microscopy, Confocal laser scanning microscopy, Multiple immunofluorescence staining, PAP method, Rapid freezing and thawing method

## Abstract

CLEM, which allows the observation of the same structure using both light and electron microscopes, is becoming increasingly popular in various fields in recent years as molecular and cell biology research deepens and experimental techniques become more sophisticated. Although CLEM is often considered a specialized technique, it has been used for 40 years to observe neuroanatomy since the era of Golgi-EM, so it does not require specialized equipment and can be applied relatively easily to various tissues and even cultured cells at a single cell level. The specific methodology is introduced here.

## Introduction

The technique of capturing the same structure firstly under a light microscope and then under an electron microscope to correlate the light and electron microscopic images has recently come to be called CLEM (correlative light and electron microscopy). CLEM, which combines the advantages of light and electron microscopy, is of great significance, and the development of equipment for this purpose is also progressing. However, CLEM already has a tradition of more than 40 years in neuroanatomy since the Golgi-electron microscopy method in the 1980s (Freund and Somogyi 1983; Takasu and Hashimoto, 1988). In neuroanatomy, it is necessary to position the target structures within the neural circuit. Therefore, electron microscope research has had to be oriented toward CLEM from the beginning. Therefore, CLEM in the field of neuroanatomy, which has been developed since such old days, does not require special equipment, and its simple method can be immediately applied to modern cutting-edge molecular and cell biology researches. In this article, the author introduces the method that has been used in our laboratory as an example of CLEM in neuroanatomy. This method can also be applied to cell and molecular visualization techniques using GFP and RFP, which are currently widely used in various fields of life science. This is because both fluorescent molecules often do not lose their antigenicity upon fixation with high concentrations of glutaraldehyde, the typical fixative used to acquire fine structures in conventional transmission EM.

## CLEM in immunohistochemical single staining using DAB

**Fig. 1:**
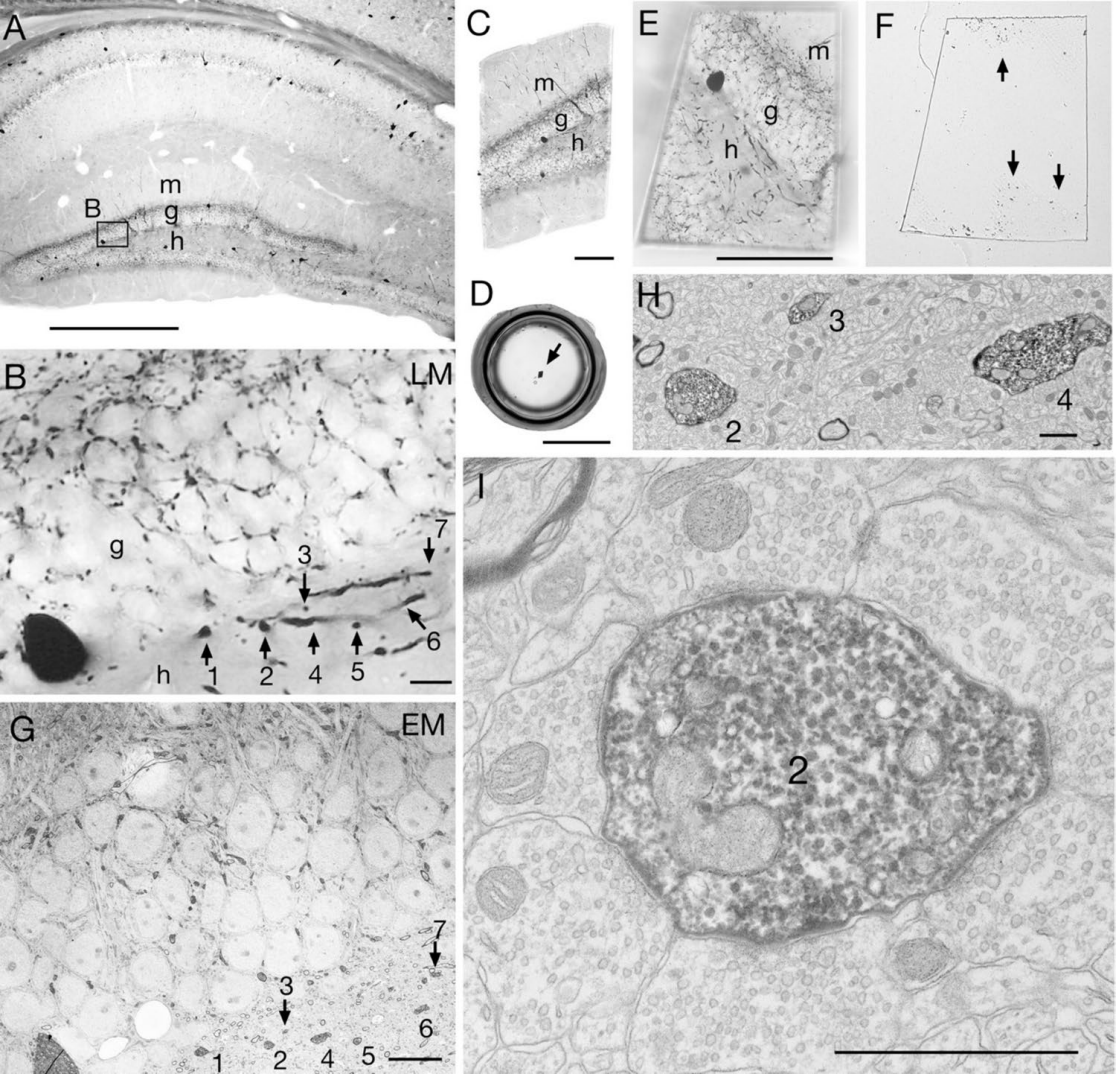
CLEM in single labeling using DAB. **A** Immunohistochemical staining for parvalbumin (PV) in the mouse hippocampus. m dentate gyrus molecular layer, g granule cell layer, h hilus. **B** An enlargement of the boxed area shown in (**B**). **B** Numbers indicate the parts of the PV-immunopositive dendrites in the hilar region. **C** The area to be observed by electron microscopy was cut out from the resin film of the tissue section. **D** The cutout part of the section was embedded in the upper surface of a cylindrical resin block (arrow). **E** The surface of the block after trimming. The immunopositive structures can be clearly observed. **F** When the semi-thin section reaches the surface of the sample, some of the immunopositive structures (arrows) appear. **G** The area corresponding to (B) was found in the serial sections by electron microscopy. **H** Low-magnification images of structures 2, 3, and 4. **I** High-magnification image of structure 2. The PV-immunopositive dendrites are surrounded by axon terminals that form asymmetric synapses. The latter are thought to originate from the mossy fiber axons of granule cells. Scale bars, 0.5 mm **A**; 10 μm **B**; 100 μm **C**; 5 mm **D**; 100 μm **E**; 10 μm **G**; 1 μm **H**, )

**Fig. 2:**
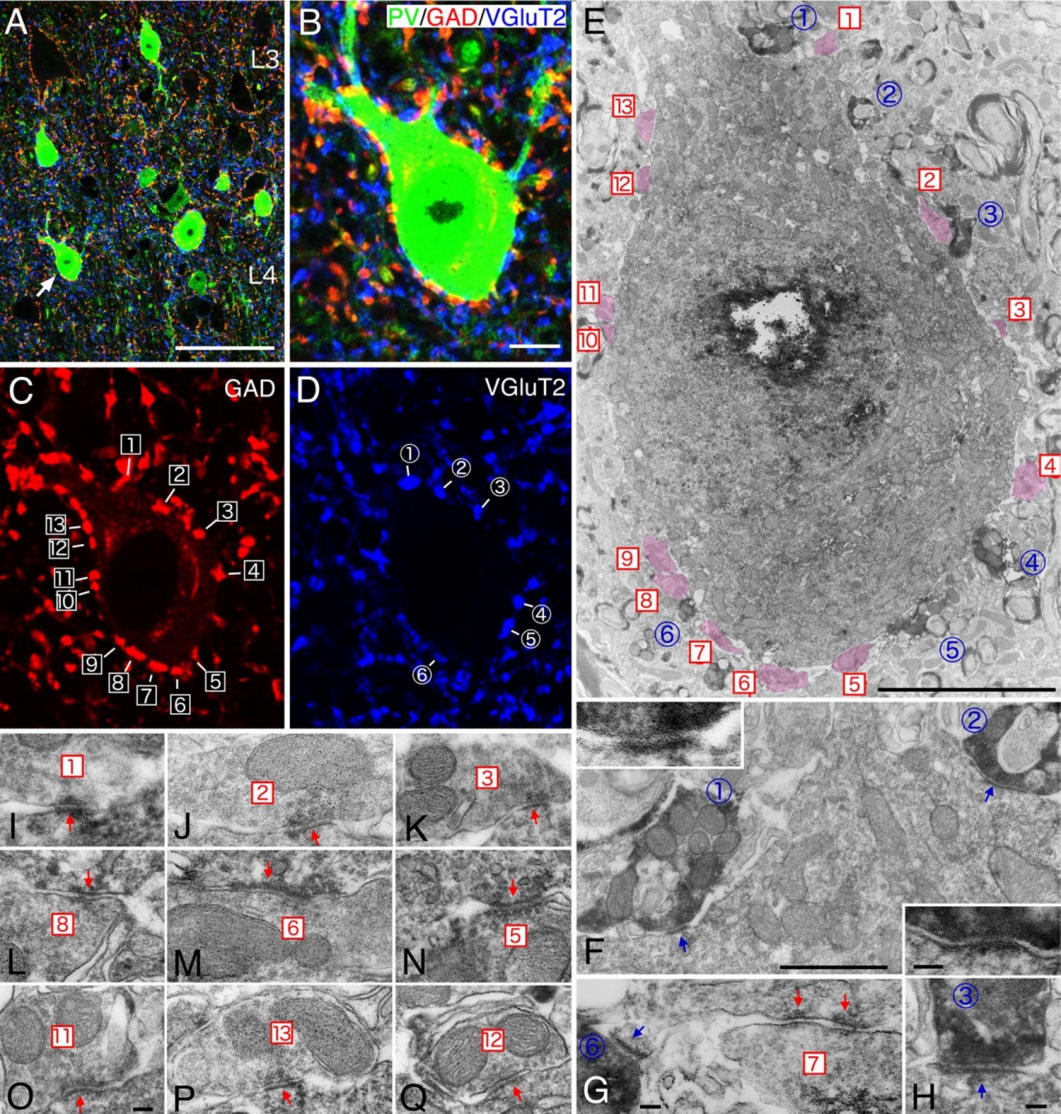
Double-labeled CLEM. **A**–**D** Immunofluorescent triple staining of cat primary visual cortex layer 4. Many GAD-positive (red) and vesicular glutamate transporter 2 (VGluT2)-positive (blue) boutons are in contact with the soma of PV-positive neurons (green). (E) PV labeling was visualized with DAB and VGluT2 labeling with DAB Nickel. Numbers shown for individual VGluT2-positive boutons in (**D**) correspond to those in (**E**). (F–H) All VGluT2-positive boutons formed asymmetric synapses with PV neurons. GAD-positive boutons in (**C**) were identified by electron microscopy based on their relative positions in (**B**-**D**), and the presence of symmetric synapses was confirmed as shown in (I-Q). Scale bars, 50 μm **A**; 5 μm **B**-**E**; 1 μm **F**; 0.1 μm (F Inset, G-Q). Reprinted from Fukuda (2017) under Elsevier’s copyright policy for open access article with CC BY-NC-ND

**Fig. 3:**
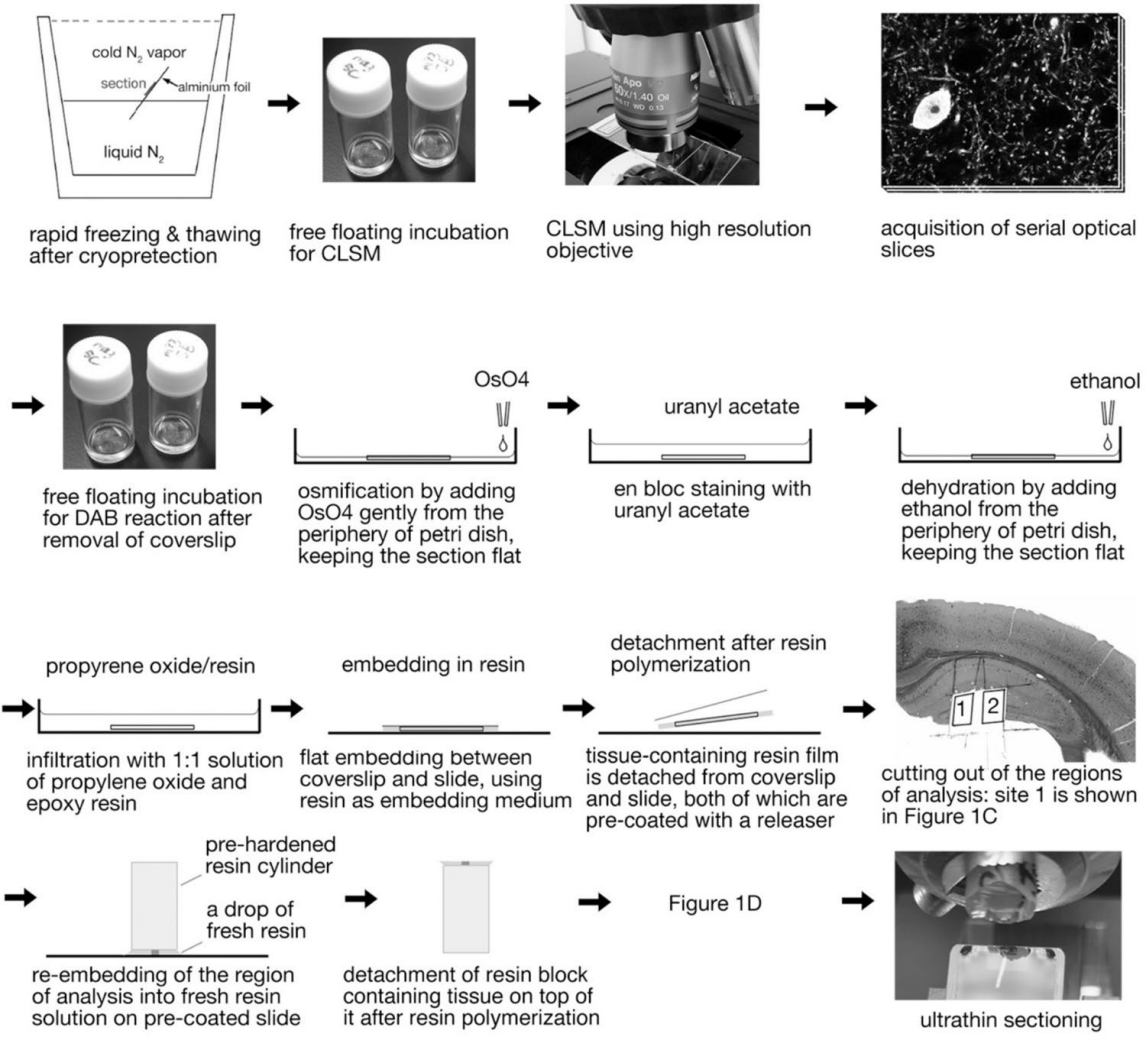
Schematic illustrations of the procedures of CLEM. Note that only conventional laboratory instruments, which are available in every EM department, are necessary to execute CLEM of high quality and sufficient reliability. Use of CLSM is very helpful but not essential; CSLM can be skipped when DAB-labeled immunostaining is conducted as an initial staining

Multiple fluorescent staining is common in current immunohistochemistry, and the method of observing the localization of a single antigen in bright field using DAB (diaminobenzidine tetrahydrochloride) is not often used recently. However, since DAB reaction products can be identified by both light and electron microscopy (Fig. 1), it is highly useful in CLEM. Furthermore, the latter half of the procedure for analyzing multi-labeled immunofluorescence specimens using confocal laser scanning light microscope (CLSM), followed by single or double EM labeling (Figs. 2, 3), is common to the method described here. The contents of each step are explained below.

### Fixative

For electron microscopy, a high concentration (2.5%) of glutaraldehyde is generally used as a fixative, but it is often not suitable for preserving antigenicity. For this reason, there are three options:

4% paraformaldehyde (PA) + 0.1% glutaraldehyde (GA) in phosphate/cacodylate buffer.

In many cases, fine structures can be observed by electron microscopy while maintaining antigenicity (Fukuda et al. 1998).

2.5% GA + 2% PA in buffer.

This is a standard fixative for electron microscopy but is not used for immunohistochemistry in general. However, in certain antigens, specific immunohistochemical staining can be obtained without any problems in antibody labeling (Fig. 1). This fixative combination preserves very fine structures, so it is worth trying the staining at the light microscope level as an initial step. Note that pretreatment with NaBH_4_, which will be described later, is essential (Kosaka et al. 1986).

iii) 4% PA in buffer.

If antigenicity is lost even with the addition of 0.1% GA, this method is unavoidable. Although fine structure is lost at a certain degree, careful preparation of each step of the specimen makes it possible to provide infallible evidence for elucidating the target issue (Fig. 2; Fukuda 2009, 2017).

### Preparation of sections for light microscopy

Vibrating microtomes (Oxford, Dosaka EM, Leica, etc.), which cut sections by slowly advancing a blade that vibrates at high frequency, are suitable for immunoelectron microscopy because they preserve fine structures even just below the cut surface of the section. Thinner sections are prone to bending and tissue damages during handling, so it is recommended to cut them at a thickness of about 60 μm.

### Pre-staining treatment

Rapid freezing and rapid thawing with liquid nitrogen.

This method is used to increase the tissue penetration of antibodies. Surfactants such as triton X wash away lipids from biologic membranes, so they cannot be used for immunoelectron microscopy. After preventing ice crystal formation during freezing by pre-treatment with a sucrose solution (12.5% ​​w/w → 25% w/w in buffer), the sections are carefully spread on thick aluminum foil using a brush under a stereomicroscope, and excess water is removed with filter paper. The jar is filled halfway with liquid nitrogen, and the tip of the aluminum foil on which the section is placed is dipped into the liquid nitrogen, while the section is instantly frozen in the vapor of the liquid nitrogen. Avoid dipping the section directly into liquid nitrogen from the beginning. Once frozen, place the entire aluminum foil in liquid nitrogen for a few seconds, then immediately thaw it in a 25% sucrose solution. This treatment improves the antibody’s tissue penetration. This is also true for conventional CLSM without EM, using detergents in incubation solution, especially when high-resolution objective is used for observations of minute structures such as bouton contacts.

NaBH_4_ treatment.

In tissues fixed with high concentrations of GA, one of the divalent aldehyde groups in GA molecule remains free in the fixed tissue, and antibody molecules bind to it, resulting in nonspecific staining. Treatment for 30 min in 1% NaBH_4_ in buffer generates hydrogen bubbles coming from inside the section, reduces the free aldehyde group, and solves the problem (Kosaka et al. 1986).

### From primary antibody reaction to DAB

The free-floating method, in which sections floating in the solution are reacted while moving them slowly on a shaker, is recommended. In CLEM, keep the temperature at 4℃ from the primary antibody to the ABC-complex. To promote antibody penetration, the sections are reacted in the primary antibody for 4 days and with the biotinylated secondary antibody overnight. Adding 0.1% sodium azide into the antibody solution is important for long-period incubation. In spite of this long incubation with primary antibody, only superficial part of the section is stained, but this is a good sign. The stain occasionally penetrates deep into the section despite absence of detergents in solution and it appears as if sections are well stained under a light microscope, but this is a bad sign. In such cases, ultrastructures, membranous integrity in particular, are often poorly preserved when viewed under an electron microscope. Rapid freezing and thawing and long antibody reactions ensure immunohistochemical staining from the surface of the section to a depth of only several micrometers, and electron microscope observation should be performed within this range. The specialized techniques for it are described later.

### From osmification to flat embedding

The floating sections are treated according to the usual method. Uranium en bloc staining is performed to increase the contrast of the membrane. This is important because the electron staining on grids after thin sectioning should be limited to a short time. In addition, the following procedure to prevent deformation of the sections during osmium treatment and ethanol dehydration is necessary. A single section is placed in a small petri dish containing 0.1 M PB, and the liquid is removed with a Pasteur pipette while maintaining the flat state of the section spreading on the dish bottom. Then, add 1% osmium tetroxide in PB little by little from the periphery of the dish. While maintaining the flat shape of the section, the amount of liquid is gradually increased until the entire section is immersed. Dehydration with ascending concentrations of ethanol is performed with a procedure similar to that in osmification. After immersion in an equal mixture of epoxy resin and propylene oxide, a small amount of new resin is placed on a slide glass, the section is placed on it, using the resin as a mounting medium, and a coverslip is placed on it. Flat embedded sections in resin are polymerized and hardened at 60 °C. The slide glass and coverslips used here are coated beforehand with a water-soluble release agent (Liquid Release Agent, Electron Microscopy Sciences).

### From light microscopy to flat re-embedding

The above methods allow good light microscopy images to be obtained after the resin has hardened (Fig. 1A, B). After photographing the target structure with a light microscope, the resin film containing the immunostained section can be easily peeled off from the coverslip and slide glass by inserting a razor blade into the gap. Cut out the target area into a small piece using a paper cutting knife (such as the OLFA Designer’s Knife 216) (Fig. 1C). The cut specimen is submerged into a drop of resin on another slide coated with a release medium, and a cylindrical block of resin that has been hardened beforehand is placed on the resin drop containing the cutout specimen. After hardening in 60 °C oven, the cylindrical block can be easily peeled off from the slide to obtain a specimen that is re-embedded on the surface of the block (Fig. 1D). The tissue image is clearly visible from the top surface of the block when illuminated from the bottom side, so the trapezoid containing the target area for observation can be obtained by trimming the unnecessary part of the tissue (Fig. 1E).

### Preparation of ultrathin sections

Before start cutting, estimate the depth from the block surface, which was faced to the slide glass before peeling, to the immunostained section using the scale on the microadjustment knob of the light microscope (the actual distance is 1.5 times the measured value).

At a first step, cut several semi-thin Sects. (0.5 μm thick) from the block surface without putting water in the boat of the diamond knife for light microscopy, and smooth the block surface. Then fill the boat with water and cut manually to the depth just above the estimated depth of embedded specimen. During this process, it is usual that water adheres to the block surface when the layer of water-soluble release agent is exposed. Wait a little while, then the block surface will dry, and continue cutting further, and you will reach the layer of the specimen that is the cutout specimen containing the immunostained section. By further cutting semi-thin sections that initially contain only resin, you will finally reach the depth at which the surface of 60 μm-thick section is exposed. Care should be taken that only a part of the 60 μm section is exposed initially (Fig. 1F), and this is the timing to replace the diamond knife for semi-thin section to that for ultrathin section. This timing can be determined by observing the change of the appearance of the block surface, picking up the semi-thin section in the boat, mounting it on slide, and checking under a light microscope. DAB-positive punctate labeling can be found at several parts (or at one corner) of the section (Fig. 1F). After aligning the edge of diamond knife for ultrathin section and the block surface carefully, a ribbon of serial ultrathin sections, which neighbor the last semi-thin section, can be obtained. Place the ribbon onto a grid with a single hole slot (VECO) that is covered with formvar membrane, or on a grid pre-treated with a strong silicon nitride membrane (SiN Window Chip, JEOL): use of these grids allows the entire specimen to be observed. The latter type can be used with a holder supplied by the manufacturer for co-relative light microscopy in CLEM.

### From electron staining to electron microscopy observation

To make it easier to distinguish the DAB reaction products from the tissue element that originally shows high electron density, electron staining should be performed in a short time. By referring to the light microscopy photograph taken in advance (Fig. 1B), the location of the corresponding immunoreactive structures and capillary vessels are used as landmarks to search for the observation targets in serial ultrathin sections (Figure G-I).

## Double-labeled CLEM

After observing a double immunofluorescent stained specimen in CLSM, one of the labeling is visualized using DAB and the other labeling using a higher-electron-density stain with DAB-Nickel to achieve double-labeled CLEM (Fukuda 2017). Furthermore, the third staining in triple fluorescent labeling can often be correctly identified with an electron microscope based on its positional relationship (Fig. 2). When using an oil immersion objective lens with a large numerical aperture, the recommended optical slice interval setting for a confocal laser microscope is about 0.15 to 0.20 μm, which corresponds to two to three consecutive electron microscope sections. Therefore, when observing consecutive ultrathin sections in EM, it is easier to identify the corresponding structures by referring to a stack file of high-magnification confocal images displayed in a laptop PC put aside. The procedure for replacing fluorescent double staining with DAB and DAB-Nickel double staining is as follows.

### Selection of secondary antibody in fluorescent double staining

One of the secondary antibodies is a biotinylated antibody, and the other is a secondary antibody labeled with a fluorescent dye. Incubate sections with only biotinylated secondary antibody overnight, followed by streptavidin-conjugated fluorescent dye and the secondary antibody conjugated with fluorescent dye of different wave length. For visualization in electron microscopy, the streptavidin-fluorophore labeling can be converted to reaction products of DAB Nickel after ABC (avidin–biotin complex, Vector) treatment, while the latter uses DAB reaction products using the PAP (peroxidase-anti peroxidase) method.

### Removal of the coverslip without tissue damage

After confocal laser microscope observations, the seals that secure the four sides of the coverslip are cut off with a razor. Place a slide glass in a petri dish containing a small amount of buffer solution and slowly shake it on a shaker. The coverslip will be released after a few hours and the section can be acquired without damage.

### ABC-DAB Nickel

Normal ABC solution can be used in the experimental condition where streptavidin-conjugated fluorescent dye “a” is bound to the biotinylated secondary antibody. The avidin–biotin complex binds to the streptavidin that already exists in the labeled tissue. When DAB Nickel is used as a coloring agent, blue-black coloring is obtained. The stained image is the same as the CLSM image obtained for “a”\". The electron density of the DAB Nickel image becomes significantly higher than that of DAB after osmium treatment, making it easily distinguishable from the DAB reaction products (Fig. 2).

### Inactivation of peroxidase by sodium azide

After DAB Nickel coloring, wash the sections with a buffer containing sodium azide to inactivate the peroxidase that is contained in ABC. Then, return the sections to the buffer that does not contain sodium azide.

### DAB coloring by PAP method

We apply the PAP method, which is a classical one and was widely used in immunostaining before the appearance of ABC method. But PAP is a useful tool for the dual CLEM.

The IgG molecule of the secondary antibody, labeled with a second fluorescent dye “b”, has two arms, only one of which bind to the antigen (here, the primary antibody), whereas another arm remains free. The PAP complex consists of peroxidase and anti-peroxidase antibody. Thus, if the anti-peroxidase antibody is made from the IgG of the same animal species as the primary antibody, the PAP complex can bind to the free arm of the secondary antibody against IgG of that species, forming a peroxidase complex, localization of which matches that of the fluorescent dye “b”". By performing DAB coloring, we can obtain a light microscopic DAB image that is the same as the confocal laser microscope image of “b”. Note that peroxidase is inactivated in a solution containing sodium azide. Thus, incubation medium containing PAP complex should be sodium azide-free.

From osmification to flat embedding

Then, prepare a specimen for electron microscopy observation using the same procedure as in the previous Sect. 2.

## Comparison with newly developed CLEM methods

A powerful new method of CLEM, the in-resin CLEM, was developed recently (Tanida et al. 2020) and is continuously being improved (Tanida et al. 2023). In-resin CLEM provides fluorescent signals in epoxy resin-embedded specimens even after procedures for preparations of EM specimens such as osmification and dehydration, both of which usually quench fluorescent signals. Observations of fluorescent labeling in thin (50 ~ 100 nm) resin section at the LM level are followed by superimposition of the signals on EM images using the same section. Thus, in-resin CLEM is more straightforward method as compared to the conventional CLEM introduced here. In addition, many other novel techniques of EM that can be combined with CLEM have been developed recently (Hayworth et a., 2014; Kislinger et al. 2023; Kubota et al. 2025). As is often the case, different experimental procedures have both advantages and disadvantages, which I discuss here so that researchers can select a particular method depending on the availability of laboratory settings, object of analysis, necessary resolution, and temporal aspects in studies.

The present method is applicable to all laboratories equipped with “conventional” experimental settings for transmission electron microscopy (TEM). Moreover, careful handling of tissues ensures deformation-free specimens and precise matching of the LM and EM images along not only x- and y- axes but also z-axis easily without the aid of software. Matching along x- and y-axes is executable only by visual inspection as shown in Fig. 1, using blood capillaries and cells surrounding target structures, in addition to DAB-labeled profiles themselves. This is as easy as matching image in toluidine-blue stained semithin section with that in ultrathin sections, a basic technique executable by all electron microscopists. The method to match images along z-direction is described above at subsection 2–7) for DAB-labeled single staining and at the first paragraph of Sect. 3 for CLSM-CLEM. However, in this respect, procedures in-resin CLEM will be simpler, because dual images are acquired from the same resin section. Another merit of in-resin CLEM is that DAB reaction products, which are essential markers in conventional CLEM, are not used for image matching. DAB reaction products more or less hide ultrastructural details. In contrast, in-resin CLEM is free from such problem. So, analysis of minute intracellular structures such as novel microstructures inside cells and subtle morphologic changes in organelles in gene-targeted cells can be performed in DAB-free in-resin CLEM. Conventional CLEM has some disadvantages in this point, but adjustment of the density of DAB reaction can settle the problem, leading to visualization of cellular junctions consisting of plasma membranes showing lipid bilayers as essential components in observations (Fukuda et al. 2006, Shigematsu et al. 2019), which cannot be discriminated at the resolution in Focused Ion Beam (FIB)- and other types of scanning electron microscope (SEM) usually adopted in in-resin and other new methods of CLEM. In conventional CLEM, use of immunogold (Asamitsu et al. 2020) and of fluoronanogold in particular (Fukuda et a., 2006, their Fig. 2) as the secondary antibody is also helpful. Fluoronanogold-conjugated antibody contains both nanogold particle and fluorophore in a single molecule, thus direct correspondence between the fluorescence signals in CLSM and ultrastructures in TEM is attained. One issue to be improved reportedly in in-resin CLEM might be the relatively rapid disappearance of fluorescent signals after embedding in resin (Tanida et al. 2023). In contrast, DAB reaction products are virtually permanent inside resin. Thus, some kinds of studies exploring cells of large size such as neurons or a broad area using CLSM at an initial step, followed by EM analysis focusing several different positions predetermined in CLSM images, which is typical in neuroanatomy, need longer period for analysis and thus suit to conventional CLEM using DAB.

One disadvantage of the conventional CLEM is the necessity of time-consuming work when 3D reconstructions are prepared using TEM images of serial ultrathin sections. Because serial ultrathin sections on grid require alignment for 3D reconstructions, we use a software Neurolucida (MicroBrightField) for alignment and tracings of objects. But efforts of preparing deformation-free specimens result in stress-free good matching of structures during the reconstruction process. FIB-SEM imaging does not need alignment, and several modern automated EM systems such as automated tape-collecting ultramicrotome, when combined with specialized applications, can collect ultrathin sections, extract structures of interest, and build 3D images, all automatically (for review, see Kislinger et al. 2023; Kubota et al 2025). Thus, these modern high-end instruments, if available, provide opportunities of EM analysis to non-specialist users of broad research fields.

Even so, the conventional CLEM is accessible to all electron microscopists worldwide, does not have limitation and can be applied in most, if not all, morphologic studies. The present article demonstrates procedures we achieved in neuroanatomical studies, but we have also conducted the same CLEM method in studies of skeletal muscles (Fujimaki et al. 2022), four-stranded gene structure in nuclei (Asamitsu et al. 2020), chondrocytes that were immunostained in paraffin sections on slides (Tanoue et al. 2018), and other diverse cells including osteoblasts after decalcification of bones, blood capillaries in diabetes mellitus model animals, cultured cells of different origins, developing neuronal processes at postnatal day 7, and chorioallantoic membrane of chick embryo (the latter 5 examples, unpublished observations in ongoing studies), all with sufficient quality. In fragile tissues such as immature brains, fixative containing high concentration of glutaraldehyde will be required in certain cases, and the present CLEM method can be applied if antigenicity is preserved in such conditions. Of note is that GFP, if expressed at sufficiently high levels, can be clearly detected unexpectedly in the CLSM using anti-GFP antibody for tissues fixed with 2.5% and even 5% of glutaraldehyde, the concentration of the original fixative of Karnovsky (1965), leading to preservation of excellent ultrastructures in fragile tissues.

## Tips

1. High resolution LM and/or CLSM beforehand is essential to interpret DAB labeling appropriately in EM. Experimental conditions for immunohistochemistry/immunofluorescence should be determined in a usual way to avoid non-specific labeling and obtain clear signals using proper controls, for which the most reliable way is the use of tissues/cells of knockout animals for control staining. If such tissues are not available, use of antibodies the specificity of which was confirmed using knockout animal tissues is recommended. Establishing proper staining conditions at the LM level is a pre-requisite for CLEM, where profiles should be the same in both initial LM observations and EM analysis (Fig. 1). Misleading by non-specific DAB labeling through lengthening of reaction time for DAB coloring can be avoided by comparing the profile with pre-observed CLSM image.
2. Pitfall in confocal imaging. Weak, possible non-specific staining can be enhanced as much as researchers want by increasing the laser power and gain of detector in CLSM. But this might lead to erroneous results. It is recommended to adjust immunofluorescence conditions so that fully bright signals can be observed under conventional epifluorescence light microscope. Then, high resolution confocal images can be acquired by adjusting the diameter of the pinhole of confocal microscope to the optimized small size pre-determined by the manufacturer.
3. Interpretation of DAB labeling. Because DAB is contained as a soluble content in the incubating medium, its reaction products adhere to not only targeted structures labeled by peroxidase-containing secondary antibody but also surrounding structures such as mitochondria. Thus, in studies that explore precise localization of molecules in a particular organelle, care should be taken in interpretation of the labeling pattern in EM, and labeling with immunogold particle might become necessary. Double labeling at the CLSM level with a marker for a particular organelle in addition to the antibody of interest is also helpful to recognize the localization of DAB reaction products accurately in EM.
4. In dual CLEM using DAB-nickel and DAB, treatment for DAB-Nickel should be conducted first, followed by inactivation of peroxidase activity with sodium azide-containing buffer and then second coloring with DAB. If DAB coloring is conducted as a first staining, this initial labeling may be intensified by DAB-Nickel solution through the possible remaining peroxidase activity, leading to difficulty in differential labeling.
5. Quantitative analysis of the labeling intensity. Because contents of DAB reaction products depend on the reaction time of a brief period with H_2_O_2_ and the labeling intensity is further enhanced greatly through osmification, quantitative analysis of labeling intensity in targeted structures such as axon terminals should be conducted in CLSM images before going to the preparation for EM observations (Fukuda et al. 1998). CLEM images using fluorophores inherently contain pixel-wide digital information regarding the labeling intensity and are thought to be less influenced by experimental conditions as compared to DAB reactions described above. The conditions of peroxidase reactions to DAB such as reaction time, pH, and temperature should be kept constant, and staining intensity and signal/noise ratio should be adjusted only though changing the concentration of primary antibodies.
6. Keeping the integrity of cellular structures. Initial fixation is the most important factor to avoid artifacts and deformation during tissue processing. When fixation and the following procedures are conducted appropriately, DAB reaction products remain inside the plasma membrane of targeted structures even if the fixative does not contain glutaraldehyde (Fig. 2).
7. Many reagents including osmium tetroxide, Nickel solution, epoxy resin, and glutaraldehyde are hazardous to both researchers and environments. So institutional regulation for handling and disposal of these materials should be strictly followed. Storage of osmium tetroxide needs particular attention, because its vapor coming from a narrow gap between the bottle and bottle cap reacts with organic substances, irrespective of all efforts to tightly seal the cap of the bottle. Thus, osmium stock solution should be stored in a refrigerator that does not contain antibodies or other biomaterials.

## Conclusion

Form and function are essentially inseparable. To perform a specific function, the existence of a unique shape that supports it is essential, whereas the function is built into the shape of the organism itself. This relationship is especially true for morphology under the electron microscope. In a sense, it is a natural course that cutting-edge research in molecular and cell biology has recently been moving back to electron microscopy. One reason is that researchers using “super-resolution” light microscopy has now become unsatisfied with its resolution but are desperate for knowing the true structure that is hidden inside monotone signals in super-resolution LM image and can be revealed only by EM. I hope that this article will be of some help in understanding and practicing old but revisited electron microscopy research in CLEM that does not require specialized, expensive equipment.
