# Correction: Introduction to the CLEM technique developed in the field of neuroanatomy

**DOI:** 10.1007/s12565-025-00893-8

**Published:** 2025-08-07

**Authors:** Takaichi Fukuda

**Affiliations:** 1 Department of Anatomy and Neurobiology, Graduate School of Medical Sciences, Kumamoto University, Kumamoto, 860-8556 Japan; 2 Center for Metabolic Regulation of Healthy Aging, Faculty of Life Sciences, Kumamoto University, Kumamoto, 860-8556 Japan; 3 Center for Clinical Laboratory, Kumamoto Rosai Hospital, 1670 Takehara-Machi, Yatsushiro, Kumamoto, 866-8533 Japan

**Correction: Anatomical Science International** 10.1007/s12565-025-00875-w

In this article, Fig. 1 was reprinted from the author’s previous article in Kaibougaku Zasshi [97 (1), 17–22, 2022, in Japanese] according to the guidelines by ICMJE.

